# Carlton C. Frederick

**Published:** 1911-06

**Authors:** Matthew D. Mann


### Carlton C. Frederick

WHEN a man like Carlton C. Frederick dies the whole community pauses to express its sorrow and regret.

Dr. Frederick was a man we can ill afford to lose for he stood for all that was best and highest in the profession which he loved so well and in the city in which he lived. A hard worker, a close student and an extraordinarily skilful operator, a good teacher and writer, a kind and helpful friend, a good fellow and a jolly companion—we shall not soon find his like again.

A graduate of one of our great universities, an ex-hospital interne, as well as a student abroad, Dr. Frederick had many advantages. These he did not waste, but improved to the utmost with the result we know so well.

Dr. Frederick's success was due not to the tricks of the charlatan or the sophistries of the grafter, but to his sterling worth. He despised all the low practices which have made some men's apparent success. When certain ethical questions were recently before the profession he circulated and asked for the signatures of the various operators of the city to a pledge drawn up by himself in which each one promised to stand for all that is best in the profession. In this he showed his own lofty position and by bringing others to it doubtless did much and lasting good. Let us honor his name for this accomplishment.

Handicapped for many years by a serious organic disease he did not let this worry him, but worked on just as hard, only saving himself by occasional trips away for rest. He was a member of both the national societies devoted to gynecology and thus counted among his friends the leading gynecologists of the country. His contributions to the transactions of both these societies were of great value and he was always listened to with respect and attention.

Of late years he had a very large and exacting practice. To this he wholly devoted himself, never refusing the heavy demands made on his time and strength. Undoubtedly he hastened his end by so doing, but he never shirked what he considered to be his duty at any cost to himself.

Such a man is an honor to his chosen profession, a credit to the race, and an example worthy to be followed.

MATTHEW D. MANN.

